# Evaluating the competence of large language models in ophthalmology clinical practice: a multi-scenario quantitative study

**DOI:** 10.3389/fcell.2025.1704762

**Published:** 2025-12-02

**Authors:** Mu-Yang Wei, Yu-Lin Li, Shu-Yan Liu, Guang-Yu Li

**Affiliations:** Department of Ophthalmology, The Second Norman Bethune Hospital of Jilin University, Changchun, China

**Keywords:** ophthalmology application, large language models, clinical accuracy, doctor-patient communication, readability analysis, sentiment analysis

## Abstract

**Background and objectives:**

A comparative evaluation of large language models (LLMs) is crucial for their application in specialized fields, such as ophthalmology. This study systematically assesses five prominent LLMs (ChatGPT 4, Claude 3 Opus, Gemini 1.5 Flash, ERNIE 3.5, and iFLY Healthcare) to quantify their performance across key clinical domains and provide evidence-based guidance for their integration.

**Methods:**

We evaluated the LLMs across three simulated ophthalmic scenarios. For clinical assistance, the models responded to 50 questions, which were assessed for accuracy, completeness, and readability. For diagnosis and treatment, models answered 375 qualification exam questions to assess clinical reasoning. For doctor-patient communication, models responded to 20 SPIKES-based scenarios, which were analyzed for emotional and social engagement.

**Results:**

In clinical assistance, Gemini 1.5 Flash demonstrated superior accuracy and completeness, while Claude 3 Opus produced the most readable text. For diagnosis and treatment, all models surpassed the passing threshold for the qualification exam, with Claude 3 Opus achieving the highest overall accuracy (81.07%). In doctor-patient communication, Gemini 1.5 Flash showed the strongest performance in positive emotional expression and social engagement.

**Conclusion:**

This study innovatively evaluates LLMs in ophthalmic practice. Gemini 1.5 Flash excels in generating accurate clinical content and engaging with patients, whereas Claude 3 Opus demonstrates exceptional clinical reasoning and readability of text. Findings validate LLMs’ clinical potential while providing evidence-based selection criteria for ophthalmic AI applications. The results establish practical foundations for optimizing ophthalmic AI model development and systematically constructing intelligent ophthalmic hospital systems.

## Graphical Abstract

**Figure:**
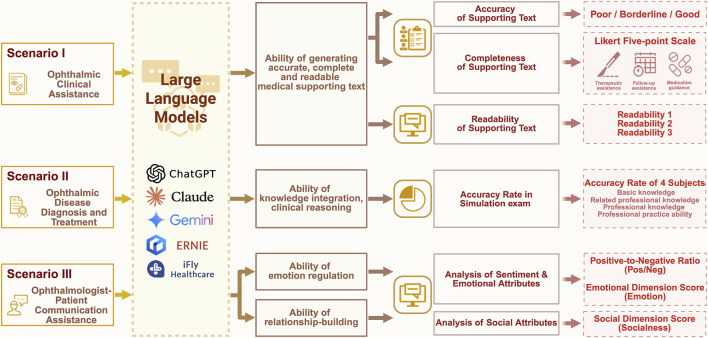
Systematic evaluation framework for LLMs in ophthalmic clinical practice across three simulated scenarios: (1) Ophthalmic Clinical Assistance scenario: Specialized question bank is designed based on guidelines to test LLMs. Ophthalmologists evaluate accuracy (Poor/Marginal/Good) and completeness (Likert 5-point scale), while automated metrics assess readability. (2) Ophthalmic Disease Diagnosis and Treatment scenario: 375 textbook questions are selected to simulate the ophthalmology qualification exam. Accuracy is measured overall and per subject (basic knowledge, related professional knowledge, professional knowledge, practice ability). (3) Ophthalmologist-Patient Communication Assistance scenario: Responses to 20 challenging dialogues guided by SPIKES protocol undergo sentiment analysis (Pos/Neg ratio, Emotion/Socialness scores).

## 1 Introduction

The concept of Artificial Intelligence (AI) was first proposed in the 1950s. In recent years, transformative breakthroughs in AI technology have emerged. Large Language Models (LLMs), leveraging deep learning methodologies, now autonomously execute diverse Natural Language Processing (NLP) tasks (Omiye et al., 2024). Trained on massive datasets, these models exhibit exceptional capabilities in language comprehension, generation, and producing human-like text that is revolutionizing medical AI applications.

Notably, ChatGPT (OpenAI) has demonstrated proficient medical knowledge application by passing the United States Medical Licensing Examination (Yaneva et al., 2024), while also exhibiting measurable empathy in patient communication and medical ethics contexts (Brin et al., 2023; Kung et al., 2023; Thirunavukarasu et al., 2023; Meng et al., 2024). With subsequent releases of models including Gemini (Google), Claude (Anthropic), ERNIE (Baidu), and iFLY Healthcare, LLMs show significant potential to augment diverse medical domains. Firstly, by synthesizing vast medical literature, LLMs generate accurate, comprehensive, and clinically actionable text (Rajabi et al., 2024; Barclay et al., 2024; Lim et al., 2023). This facilitates rapid integration of cutting-edge research into diagnostic and therapeutic decision-making. Secondly, LLMs demonstrate emerging utility in preliminary disease assessment (Delsoz et al., 2023; Madadi et al., 2023), analyzing patient histories to propose differential diagnoses and evidence-based treatment strategies. Thirdly, by leveraging sophisticated emotion regulation and relational capabilities (Ayers et al., 2023; Yeo et al., 2023), LLMs craft context-appropriate communication templates that strengthen clinician-patient rapport and foster therapeutic trust. As large language models are officially multilingual and demonstrate strong competence in Mandarin comprehension and generation, this study investigates their effectiveness in Chinese ophthalmic clinical contexts by systematically evaluating five representative LLMs (ChatGPT 4, Claude 3 Opus, Gemini 1.5 Flash, ERNIE 3.5, and iFLY Healthcare) across three real-world ophthalmology practice scenarios.

## 2 Methods

### 2.1 Ophthalmic clinical assistance scenario simulation and evaluation methodology

Fifty professional questions were designed to simulate ophthalmic clinical assistant scenarios, following authoritative Chinese ophthalmic guidelines and expert consensus. A detailed list of all Chinese ophthalmic guidelines and expert consensuses used for question design is provided in Supplementary Table S1. These questions cover common ophthalmic diseases across eight categories (cataracts, glaucoma, refractive errors, age-related macular degeneration, diabetic retinopathy, retinal vein occlusion, ocular surface diseases, orbital diseases, and ocular trauma), ensuring their practical value. Corresponding reference answers were collected based on the latest guidelines and consensus, enabling specialists to assess the quality of text generated by LLMs.

From April 20 to 30 April 2024, ChatGPT 4, Claude 3 Opus, Gemini 1.5 Flash, ERNIE 3.5, and iFLY Healthcare were tested by submitting each question once in a new dialogue session. All queries and prompts employed in this study were originally composed and administered in Mandarin Chinese. Responses generated by LLMs were verbatim transcribed and archived in Microsoft Excel spreadsheets (Microsoft Corporation, Redmond, WA, United States).

The 50 questions were categorized into eight disease-specific groups. For each disease category, three ophthalmology residents specializing in the corresponding subspecialty independently rated the LLM-generated responses using the standardized evaluation forms. A blinded design was adopted, in which the evaluation materials contained only the question, the generated response, and the reference answer, while concealing the identity of the language model to ensure unbiased assessment.

Fleiss’ Kappa analysis was applied to assess inter-rater reliability for accuracy evaluations, while Kendall’s W analysis was conducted for completeness assessments. Accuracy grading criteria included three tiers: Poor (text contains substantial inaccuracies that may misguide clinicians or harm patients), Borderline (minor factual errors unlikely to cause clinical harm), and Good (error-free content). Completeness was measured using a 5-point Likert scale (1: Not comprehensive; 2: Marginally comprehensive; 3: Moderately comprehensive; 4: Comprehensive; 5: Extremely comprehensive), with responses graded Poor in accuracy excluded from completeness analysis. For statistical analysis, the three results from three ophthalmologists were summed to yield a total Completeness Score ranging from 3 to 15 for each item.

Readability refers to the ease with which a text can be read and understood. It is a key metric for assessing the quality of medical assistance texts. In this study, readability was evaluated using the readability function in the Cntext-2.1.3 package within the Stata module (Deng and Nan, 2025). Three main outcome indicators were used. The first is the average number of characters per clause (Readability1, R1). The second is the proportion of adverbs and conjunctions in each sentence (Readability2, R2). The third indicator, Readability3 (R3), is a composite score calculated by combining the first two indicators. This metric is based on the Fog Index used in English readability analysis (Gunning, 1952), as in Equation 1. Higher values for these indicators suggest greater linguistic complexity and lower readability (Xu et al., 2021).

$$\mathrm{Readability}3=\left(\mathrm{Readability}1+\mathrm{Readability}2\right)\times 0.5$$ (1)

### 2.2 Ophthalmic disease diagnosis and treatment scenario simulation and evaluation methodology

The intermediate ophthalmology qualification examination in China comprises four domains: Basic Knowledge, Related Professional Knowledge, Professional Knowledge, and Professional Practice Ability, designed to assess physicians’ ophthalmic theoretical knowledge and clinical skills comprehensively. This study randomly selected 375 choice questions from the “2024 Ophthalmology Synchronized Exercise” (People’s Medical Publishing House Co., Ltd), with question distribution strictly adhering to the examination blueprint: 100 questions each from Basic Knowledge (Subject 1), Related Professional Knowledge (Subject 2), and Professional Knowledge (Subject 3), and 75 questions from Professional Practice Competency (Subject 4).

From November 30 to 15 December 2024, ChatGPT 4, Claude 3 Opus, Gemini 1.5 Flash, ERNIE 3.5, and iFLY Healthcare were evaluated by submitting each question in a new and independent dialogue session to avoid contextual bias. All questions were originally presented to each model in Mandarin Chinese. Input templates included a prompt statement (“Help me select the single best answer” for single-choice questions; “Select the most appropriate n options” for multiple-choice questions, where n indicates the correct answer count), the question stem, and response options (each option separated by line breaks). (The English prompt statements displayed in this section are faithful translations of the original Mandarin templates, provided solely to illustrate the prompt structure and were not used in any experimental interaction). If the model’s response does not match the correct choice, a second request is submitted in the same dialogue box, and the result is noted. The accuracy of each model’s final answer serves as the outcome.

### 2.3 Ophthalmologist-patient communication assistance scenario simulation and evaluation

This study curated 20 clinically challenging patient queries through questionnaire analysis to simulate physician-patient communication scenarios. A structured questionnaire titled “Complex Components of Ophthalmic Doctor-Patient Communication” (ID: 295697760) was distributed via the Wenjuanxing platform (Changsha Ranxing Information Technology Co., Ltd.) to ophthalmologists, comprising two sections: (1) a choice question (“Select the most difficult communication task in clinical practice based on your experience: A. The complex pathogenesis of the disease and the diagnostic process; B. The limitations of treatment, potential adverse effects, and prognosis; C. The impact of the disease on patients’ quality of life and strategies to alleviate negative emotions; D. The financial burden of treatment and the support provided by health insurance policies.”) and (2) an open-ended question (“Describe the most challenging communication issue requiring AI assistance.”).

From December 15 to 20 December 2024, ChatGPT 4, Claude 3 Opus, Gemini 1.5 Flash, ERNIE 3.5, and iFLY Healthcare were evaluated by submitting each question once in new dialogue sessions. All model inputs and responses were likewise conducted entirely in Mandarin Chinese. Input templates integrated the SPIKES communication framework (Baile et al., 2000) to constrain the model to generate text in a unified structure. The prompt was: “As a Chinese ophthalmologist, respond to the patient’s query using the SPIKES protocol (S: Setting, P: Perception, I: Invitation/Information, K: Knowledge, E: Empathy, S: Summary/Strategy) to enhance communication efficiency while demonstrating empathy. Question: [text].” Model-generated responses were recorded, processed by removing non-functional annotations (e.g., protocol phase labels), and archived in Microsoft Excel (Microsoft Corporation, Redmond, WA, United States). The English prompts provided in this manuscript serve only as illustrative translations of the original Mandarin prompts and were not used in any phase of model testing.

Sentiment analysis was conducted using the Cnsenti library’s sentiment_calculate function (Deng, 2025), quantifying positive (Pos) and negative (Neg) sentiment lexemes in Chinese texts. The database integrates the HowNet lexicon and the sentiment ontology developed by the Information Retrieval Laboratory of Dalian University of Technology (Xu et al., 2008). The algorithm incorporated intensity adverb modulation and negation reversal detection to improve analytical precision. The Pos/Neg ratio served as the primary metric to mitigate text-length bias. In addition to polarity-based analysis, the study applies the sentiment_by_valence function from the Stata module of the Cntext-2.1.3 library. This function extracts two semantic scores: Socialness and Emotion (Deng and Nan, 2025). These scores are derived from the Six Semantic Dimension Database (SSDD), which provides subjective ratings for 17,940 frequently used Chinese words across six semantic dimensions: vision, motion, socialness, emotion, time, and space (Wang et al., 2023). These dimensions are designed to reflect how lexical meanings are represented and processed in the human brain. The Emotion dimension reflects the degree to which a word is associated with positive or negative affect. The Socialness dimension measures the extent to which a word relates to interpersonal relationships or social interaction.

## 3 Results

### 3.1 Evaluation of models in the ophthalmic clinical assistance scenario

#### 3.1.1 Inter-rater reliability analysis for accuracy and completeness assessments

In the assessment of accuracy, substantial inter-rater agreement was observed among the three independent evaluators across seven disease-specific subgroups, including cataract, refractive error, age-related macular degeneration, glaucoma, diabetic retinopathy, retinal vein occlusion, and orbital and ocular trauma (κ = 0.617–0.814). Only the ocular surface disease subgroup showed moderate agreement (κ = 0.310) (Table 1).

**TABLE 1:** Fleiss Kappa reliability analysis of accuracy evaluators and Kendall’s W reliability analysis of completeness evaluators.

Group	Accuracy evaluators		Completeness evaluators	
	*P*	κ	*P*	W
Cataract (n = 6)	<0.001	0.617	<0.001	0.846
Refractive error (n = 8)	<0.001	0.814	<0.001	0.762
Age-related macular degeneration (n = 6)	<0.001	0.746	0.002	0.645
Glaucoma (n = 5)	<0.001	0.725	<0.001	0.829
Central retinal vein occlusion (n = 5)	<0.001	0.802	<0.001	0.767
Diabetic retinopathy (n = 5)	<0.001	0.720	<0.001	0.772
Ocular surface diseases (n = 6)	0.003	0.310	<0.001	0.785
Ocular trauma and orbital diseases (n = 9)	<0.001	0.785	0.001	0.612

Regarding completeness assessment, consistently high levels of inter-rater reliability were achieved across all subspecialty subgroups, as indicated by Kendall-W’s coefficient of concordance (W = 0.612–0.846), suggesting robust internal consistency and rating stability (Table 1).

#### 3.1.2 Comparative accuracy analysis across the clinical support scenario

Professional ophthalmology evaluations revealed no responses graded as “*Poor*” across all LLMs. Significant inter-model performance disparities were observed (χ^2^ = 25.124, P < 0.001, Cramér’s V = 0.183). Although the effect size was small to moderate, the finding remains clinically meaningful. The proportion of “*Good*” ratings was highest for Gemini 1.5 Flash (80.7%) and lowest for iFLY Healthcare (60.0%), representing a 20.7% difference between the two models.

**FIGURE 1:**
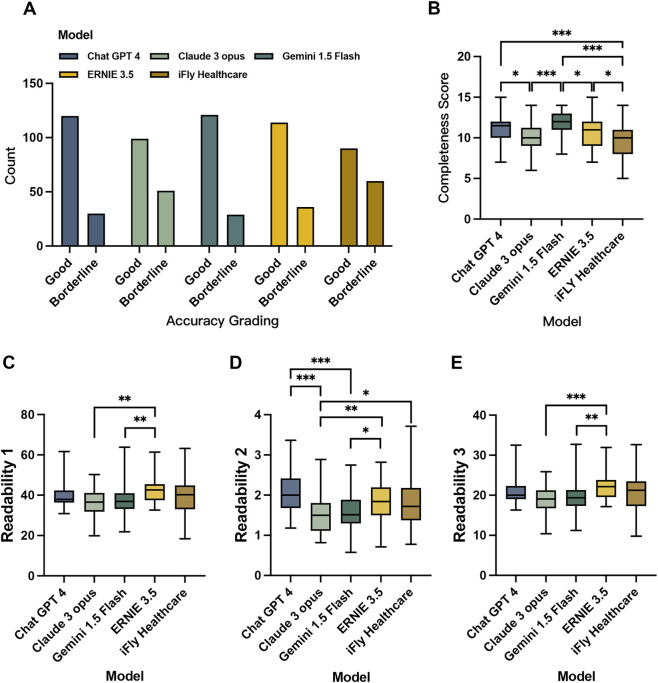
Accuracy grading **(A)**, completeness score **(B)** and readability **(C–E)** of LLM-generated text in the ophthalmic clinical assistance scenario.

Bonferroni *post hoc* analysis revealed that Gemini 1.5 Flash was statistically superior to Claude 3 Opus (*P* = 0.041, φ = 0.166) and iFLY Healthcare (*P* < 0.001, φ = 0.226) in terms of accuracy. Concurrently, ChatGPT 4 (*P* = 0.002, φ = 0.218) and ERNIE 3.5 (*P* = 0.030, φ = 0.172) exhibited significant accuracy advantages over iFLY Healthcare (Table 2; Figure 1A).

**TABLE 2:** Comparative analysis of large language model accuracy grading and completeness scores.

LLM1	LLM2	Accuracy grading			Completeness scores		
		χ^2^	*P*	φ	t	*P*	Cohen’s d
ChatGPT 4	Claude 3 Opus	7.458	0.063	0.158	2.749	0.050	0.562
ChatGPT 4	Gemini 1.5 Flash	0.021	8.845	0.008	−1.869	0.337	0.399
ChatGPT 4	ERNIE 3.5	0.699	4.030	0.048	0.880	0.904	0.167
ChatGPT 4	iFLY healthcare	14.286	0.002	0.218	4.178	<0.001	0.777
Claude 3 Opus	Gemini 1.5 Flash	8.25	0.041	0.166	−4.618	<0.001	1.055
Claude 3 Opus	ERNIE 3.5	3.643	0.563	0.110	−1.869	0.337	0.374
Claude 3 Opus	iFLY healthcare	1.158	2.818	0.062	1.429	0.609	0.280
Gemini 1.5 Flash	ERNIE 3.5	0.962	3.266	0.057	2.749	0.050	0.572
Gemini 1.5 Flash	iFLY healthcare	15.352	<0.001	0.226	6.048	<0.001	1.230
ERNIE 3.5	iFLY healthcare	8.824	0.030	0.172	3.299	0.010	0.601

#### 3.1.3 Comparative completeness analysis across the clinical support scenario

Significant differences emerged in completeness scores of LLM-generated clinical assistance texts (F = 11.12, *P* < 0.001, η^2^ = 0.154), with Gemini 1.5 Flash achieving the highest mean score (11.82 ± 1.51) and iFLY Healthcare the lowest (9.62 ± 2.03). Tukey *post hoc* pairwise comparisons demonstrated Gemini 1.5 Flash’s superiority over Claude 3 Opus (*P* < 0.001, d = 1.055), ERNIE 3.5 (*P* = 0.050, d = 0.572), and iFLY Healthcare (*P* < 0.001, d = 1.230). ChatGPT 4 exhibited significantly higher completeness than Claude 3 Opus (*P* = 0.050, d = 0.562) and iFLY Healthcare (*P* < 0.001, d = 0.777), while ERNIE 3.5 outperformed iFLY Healthcare (*P* = 0.01) in completeness metrics. 60% of the pairwise comparisons demonstrated statistical significance with at least a moderate effect size, indicating that the differences in completeness scores among LLMs are not only statistically significant but also of substantial clinical relevance (Table 2; Figure 1B).

#### 3.1.4 Comparative readability analysis across the clinical support scenario

Statistically significant variations were observed in the Kruskal–Wallis analysis of Readability 1 (R1) (χ^2^ = 20.13, *P* < 0.001, ε^2^ = 0.066). Claude 3 Opus demonstrates the lowest mean R1 value of 36.56, indicating the shortest mean sentence length. Simultaneously, ERNIE 3.5 exhibited the highest R1 value of 42.59, reflecting the longest mean sentence length. Dwass-Steel-Critchlow-Fligner (DSCF) test *post hoc* comparisons showed Claude 3 Opus (*P* = 0.001, r = 0.546) and Gemini 1.5 Flash (*P* = 0.002, r = 0.532) had significantly lower R1 values than ERNIE 3.5, with both comparisons demonstrating large effect sizes (r > 0.5), indicating substantial practical significance (Table 3; Figure 1C).

**TABLE 3:** Comparative analysis of large language model readability.

LLM1	LLM2	Readability1			Readability2			Readability3		
		W	*P*	r	W	*P*	r	W	*P*	r
ChatGPT 4	Claude 3 Opus	−3.139	0.172	0.314	−7.461	<0.001	0.746	−3.554	0.088	0.355
ChatGPT 4	Gemini 1.5 Flash	−2.662	0.327	0.266	−6.300	<0.001	0.63	−2.944	0.228	0.294
ChatGPT 4	ERNIE 3.5	3.325	0.129	0.333	−2.522	0.384	0.252	2.974	0.219	0.297
ChatGPT 4	iFLY healthcare	−0.058	1.000	0.006	−3.102	0.182	0.31	−0.224	1.000	0.022
Claude 3 Opus	Gemini 1.5 Flash	0.385	0.999	0.038	1.663	0.765	0.166	0.531	0.996	0.053
Claude 3 Opus	ERNIE 3.5	5.460	0.001	0.546	5.374	0.001	0.537	5.577	<0.001	0.558
Claude 3 Opus	iFLY healthcare	2.194	0.529	0.219	4.209	0.024	0.421	2.311	0.476	0.231
Gemini 1.5 Flash	ERNIE 3.5	5.323	0.002	0.532	4.037	0.035	0.404	5.372	0.001	0.537
Gemini 1.5 Flash	iFLY healthcare	1.740	0.734	0.174	2.774	0.285	0.277	1.813	0.703	0.181
ERNIE 3.5	iFLY healthcare	−2.627	0.341	0.263	−0.912	0.968	0.091	−2.652	0.331	0.265

Significant inter-model disparities were detected in Readability 2 (R2) (χ^2^ = 37.27, *P* < 0.001, ε^2^ = 0.136), representing a medium-to-large effect size, with model type explaining the greatest proportion of variance among all readability metrics. Claude 3 Opus displayed the lowest R2 values, contrasting with ChatGPT 4’s highest R2 values. Claude 3 Opus showed lower R2 values than ChatGPT 4 (*P* < 0.001, r = 0.746), ERNIE 3.5 (*P* = 0.001, r = 0.537), and iFLY Healthcare (*P* = 0.024, r = 0.421). Gemini 1.5 Flash also had lower R2 values compared to ChatGPT 4 (*P* < 0.001, r = 0.630) and ERNIE 3.5 (*P* = 0.035, r = 0.404). Notably, the comparisons between ChatGPT 4 and both Claude 3 Opus and Gemini 1.5 Flash yielded large effect sizes (r > 0.6), indicating that ChatGPT 4’s superiority in R2 performance holds meaningful clinical relevance (Table 3; Figure 1D).

For Readability 3 (R3) assessment (χ^2^ = 21.16, *P* < 0.001, ε^2^ = 0.070), a medium effect size was observed. Claude 3 Opus achieved the lowest mean R3 values, whereas ERNIE 3.5 showed the highest values, reflecting the greatest readability complexity. Both Claude 3 Opus (*P* < 0.001, r = 0.558) and Gemini 1.5 Flash (*P* = 0.001, r = 0.537) exhibited significantly lower R3 values compared to ERNIE 3.5. Both comparisons showed large effect sizes (*r* > 0.5), indicating strong differences in readability performance (Table 3; Figure 1F).

### 3.2 Evaluation of models in the ophthalmic disease diagnosis and treatment scenario

Significant inter-model variations in correctness rates were observed in the Chi-square test across the complete set of 375 choice test items (χ^2^ = 24.862, *P* < 0.001, Cramér’s V = 0.115), with Claude 3 Opus demonstrating the highest overall correctness rate and ChatGPT 4 showing the lowest performance. Although statistically significant, the overall effect size was small, suggesting modest practical differences between models. Bonferroni *post hoc* analysis confirmed Claude 3 Opus’s total score was significantly superior to ChatGPT 4 (*P* < 0.001, φ = 0.225), Gemini 1.5 Flash (*P* = 0.007, φ = 0.176), and ERNIE 3.5 (*P* = 0.020, φ = 0.160).

**FIGURE 2:**
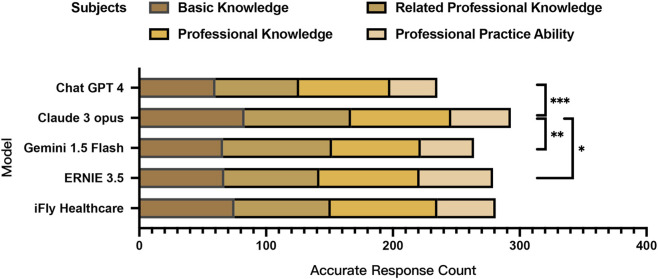
Simulation test scores by subject for large language models in the ophthalmic disease diagnosis and treatment scenario.

Further stratification by examination modules revealed distinct performance patterns. In the Basic Knowledge subject (Subject 1) (*P* = 0.004, Cramér’s V = 0.175), the Professional Knowledge subject (Subject 2) (*P* = 0.006, Cramér’s V = 0.171), and the Professional Practice Ability subject (Subject 4) (*P* = 0.009, Cramér’s V = 0.191), statistically significant inter-model differences were detected in Chi-square tests. Claude 3 Opus achieved the highest accuracy in Subject I, significantly outperforming ChatGPT 4 (*P* = 0.003, φ = 0.360). For Subject 2, Gemini 1.5 Flash attained peak performance while ChatGPT 4 recorded the lowest accuracy, with Claude 3 Opus (*P* = 0.033, φ = 0.294) and Gemini 1.5 Flash (*P* = 0.009, φ = 0.331) demonstrating statistically significant superiority over ChatGPT 4. In Subject 3, iFLY Healthcare emerged as the top performer with the highest correctness rate, whereas Gemini 1.5 Flash showed the lowest performance. Claude 3 Opus maintained its leading position in Subject 4, exhibiting a moderate-to-large effect size compared with iFLY Healthcare (*P* = 0.004, φ = 0.411) (Tables 4 and 5; Figure 2).

**TABLE 4:** Simulated examination overall and subspecialty performance.

Category	Classification	ChatGPT 4	Claude 3 Opus	Gemini 1.5 Flash	ERNIE 3.5	iFLY Healthcare	χ^2^	*P*	Cramér’s V
Overall (n = 375)	Correct	244	304	264	268	272	24.862	<0.001	0.115
	Incorrect	131	71	111	107	103			
Subject 1 (n = 100)	Correct	60	83	66	67	75	15.239	0.004	0.175
	Incorrect	40	17	34	33	25			
Subject 2 (n = 100)	Correct	66	84	86	75	76	14.589	0.006	0.171
	Incorrect	34	16	14	25	24			
Subject 3 (n = 100)	Correct	72	79	70	79	84	7.341	0.119	0.121
	Incorrect	28	21	30	21	16			
Subject 4 (n = 75)	Correct	46	58	42	47	37	13.606	0.009	0.191
	Incorrect	29	17	33	28	38			

**TABLE 5:** Comparison of total and Subspecialty simulation test scores.

LLM1	LLM2	Total (n = 375)			Subject 1 (n = 100)			Subject 2 (n = 100)			Subject 4 (n = 75)		
		χ^2^	*P* (Bonferroni)	φ	χ^2^	*P* (Bonferroni)	φ	χ^2^	*P* (Bonferroni)	φ	χ^2^	*P* (Bonferroni)	φ
ChatGPT 4	Claude 3 Opus	24.391	<0.001	0.255	12.980	0.003	0.360	8.640	0.033	0.294	4.515	0.336	0.246
ChatGPT 4	Gemini 1.5 Flash	2.440	1.183	0.081	0.772	3.795	0.088	10.965	0.009	0.331	0.440	5.072	0.077
ChatGPT 4	ERNIE 3.5	3.545	0.597	0.097	1.057	3.039	0.103	1.947	1.629	0.140	0.028	8.664	0.019
ChatGPT 4	iFLY healthcare	4.870	0.273	0.114	5.128	0.235	0.226	2.428	1.192	0.156	2.185	1.394	0.171
Claude 3 Opus	Gemini 1.5 Flash	11.608	0.007	0.176	7.606	0.058	0.276	0.157	6.921	0.040	7.680	0.056	0.320
Claude 3 Opus	ERNIE 3.5	9.547	0.020	0.160	6.827	0.090	0.261	2.485	1.149	0.158	3.841	0.500	0.226
Claude 3 Opus	iFLY healthcare	7.663	0.056	0.143	1.929	1.649	0.139	2.000	1.573	0.141	12.660	0.004	0.411
Gemini 1.5 Flash	ERNIE 3.5	0.103	7.477	0.017	0.022	8.809	0.015	3.854	0.496	0.196	0.691	4.059	0.096
Gemini 1.5 Flash	iFLY healthcare	0.418	5.177	0.033	1.947	1.629	0.140	3.249	0.715	0.180	0.669	4.136	0.094
ERNIE 3.5	iFLY healthcare	0.106	7.450	0.017	1.554	2.125	0.125	0.027	8.694	0.016	2.706	1.000	0.208

### 3.3 Evaluation of models in the ophthalmologist-patient communication assistance scenario

This investigation utilized a questionnaire survey aimed at ophthalmologists with a minimum of 2 years of clinical experience, gathering 60 valid questionnaires to identify complex aspects of physician-patient communication in ophthalmic practice. Questionnaire recovery and response statistics are shown in Supplementary Table S2. Based on response distributions, 20 clinically representative communication test items were designed proportionally.

The evaluation of emotional generation capacity showed no significant differences between models in the ratios of positive to negative sentiment words (Pos/Neg) or emotional dimension values (Emotion). Descriptively, Gemini 1.5 Flash demonstrated the highest Pos/Neg ratio, indicating the most positive responses, while Claude 3 Opus showed the lowest ratio. In emotional dimension assessment, Gemini 1.5 Flash attained the highest Emotion values, representing the strongest affective expression, in contrast to ChatGPT 4’s minimal Emotion values (Table 6).

**TABLE 6:** Statistical analysis of text sentiment polarity and emotional/socialness dimension.

Polarity/Dimension	ChatGPT 4 (n = 20)	Claude 3 Opus (n = 20)	Gemini 1.5 Flash (n = 20)	ERNIE 3.5 (n = 20)	iFLY Healthcare (n = 20)	F/χ^2^	*P*
Pos/Neg	2.29 (1.72, 2.99)	1.95 (1.47, 2.84)	2.82 (1.78, 4.49)	2.25 (1.45, 3.22)	2.17 (1.65, 3.55)	3.36	0.499
Emotion	56.01 ± 16.53	65.00 ± 38.30	72.45 ± 33.85	72.04 ± 28.36	69.17 ± 29.55	1.01	0.406
Socialness	516.50 (489.33, 575.25)	519.10 (452.86, 570.45)	774.67 (705.11, 813.73)	701.67 (666.73, 779.40)	621.17 (548.29, 724.40)	41.66	<0.001

**FIGURE 3:**
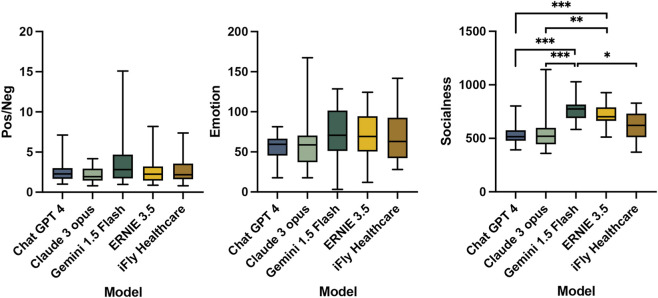
Sentiment polarity and emotional/socialness dimension analysis of LLM-generated text in the ophthalmologist-patient communication assistance scenario.

Assessment of relation-building competence through socialness dimension values demonstrated significant inter-group differences (χ^2^ = 41.66, *P* < 0.001, η^2^ = 0.396). Gemini 1.5 Flash generated text with the highest Socialness values, indicating superior social engagement capacity. Claude 3 Opus exhibited the lowest values. DSCF test *post hoc* analysis confirmed Gemini 1.5 Flash’s socialness values significantly exceeded those of ChatGPT 4 (*P* < 0.001, r = 1.119), Claude 3 Opus (*P* < 0.001, r = 0.986), and iFLY Healthcare (*P* = 0.015, r = 0.702), all demonstrating large to very large effect sizes. Additionally, ERNIE 3.5 demonstrated significantly higher socialness scores compared to ChatGPT 4 (*P* < 0.001, r = 0.986) and Claude 3 Opus (*P* = 0.001, r = 0.853), also with large effect sizes (Table 7; Figure 3).

**TABLE 7:** Text socialness dimension comparison.

LLM1	LLM2	W	*P*	r
ChatGPT 4	Claude 3 Opus	−0.440	0.998	0.070
ChatGPT 4	Gemini 1.5 Flash	7.077	<0.001	1.119
ChatGPT 4	ERNIE 3.5	6.236	<0.001	0.986
ChatGPT 4	iFLY healthcare	3.366	0.121	0.532
Claude 3 Opus	Gemini 1.5 Flash	6.236	<0.001	0.986
Claude 3 Opus	ERNIE 3.5	5.394	0.001	0.853
Claude 3 Opus	iFLY healthcare	2.869	0.252	0.454
Gemini 1.5 Flash	ERNIE 3.5	−1.874	0.676	0.296
Gemini 1.5 Flash	iFLY healthcare	−4.437	0.015	0.702
ERNIE 3.5	iFLY healthcare	−2.640	0.336	0.417

## 4 Discussion

This study conducted a comprehensive evaluation of the clinical application capabilities of five large language models (ChatGPT 4, Claude 3 Opus, Gemini 1.5 Flash, ERNIE 3.5, and iFLY Healthcare) in ophthalmic practice through simulated clinical scenarios: ophthalmic clinical assistance, preliminary ophthalmic disease diagnosis and treatment, and assistance for ophthalmologist-patient communication. The findings demonstrate varying performance levels across models in these clinical contexts. The evaluated LLMs exhibited measurable clinical auxiliary capacities, including medical supplementary material generation, ophthalmic knowledge integration, clinical reasoning capabilities, emotional regulation, and relation-building in text generation. However, potential application risks requiring clinical vigilance were identified, particularly instances of harmful content and factual inaccuracies in specific model responses. Subsequent sections will provide a detailed discussion addressing these critical findings within the framework of ophthalmic clinical practice requirements.

### 4.1 Comparison with existing ophthalmic LLM studies

The application of LLMs in ophthalmology has garnered increasing research interest. Existing literature predominantly focuses on qualification exam performance and subspecialty-specific applications. While these studies have established the foundational competence of earlier LLMs, significant gaps remain in comprehensively evaluating their capabilities across diverse clinical applications.

Previous international studies have predominantly employed single-scenario evaluations. Most commonly, researchers have utilized ophthalmic qualification examination question banks to assess model performance and compare it with human benchmarks, such as Basic and Clinical Science Course (BCSC) self-assessment programs (Cai et al., 2023; Teebagy et al., 2017) and The Royal College of Ophthalmologists (FRCOphth) sample questions (Fowler et al., 2024; Raimondi et al., 2023). Alternatively, studies have focused on specific subspecialty question-and-answer scenarios, such as thyroid eye disease (TED) management (Rajabi et al., 2024; Ali, 2023) or pediatric myopia counseling (Lim et al., 2023; Nikdel et al., 2024). Research on glaucoma (Delsoz et al., 2023; Kianian et al., 2024a), uveitis (Rojas-Carabali et al., 2024a; Rojas-Carabali et al., 2024b; Kianian et al., 2024b), and neuro-ophthalmology (Madadi et al., 2023; Tao et al., 2024; Tailor et al., 2025) has primarily concentrated on diagnostic accuracy or patient education text generation within narrow clinical contexts. While these investigations have validated LLM accuracy at a certain knowledge level, they have not extended to broader or more complex clinical environments.

Some studies have explored readability using tools like Flesch-Kincaid scores (Kianian et al., 2024b; Balas et al., 2024; Eid et al., 2024; Momenaei et al., 2023) or attempted to measure empathy through EMPATHY scales or specialized quality assessment tools (Brin et al., 2023; Ayers et al., 2023; Webb, 2023), a unified, multifaceted evaluation framework integrating cognitive, linguistic, and emotional dimensions has been lacking. Previous ophthalmic LLM studies focusing exclusively on factual correctness have provided limited insight into the comprehensive competencies required for clinical practice.

Furthermore, prior research has primarily compared one to two models (typically GPT versions or ChatGPT versus Google Bard/Gemini) in English-language contexts, with limited systematic inclusion of other advanced models. Additionally, western-trained models show significantly degraded performance in non-English environments (Sakai et al., 2023), highlighting critical limitations in cross-linguistic generalizability. Most existing studies have been conducted exclusively in English-language contexts, with insufficient evaluation of LLM performance in Chinese medical terminology and clinical communication.

Our study addresses these critical limitations through three synergistic innovations. First, in evaluation scenarios, we propose a multi-scenario framework that transcends question-bank testing paradigms, systematically simulating authentic clinical environments. Second, in assessment methodology, we incorporate emotional intelligence and linguistic quality indicators into systematic evaluation within a unified framework. Third, in model diversity, we systematically evaluated five platforms, including ChatGPT-4, Claude 3 Opus, Gemini 1.5 Flash, and Chinese-native LLMs (ERNIE 3.5 and iFLY Healthcare) within Chinese clinical contexts. This represents the first systematic cross-platform comparison in ophthalmology, providing critical data on global versus localized model performance and addressing documented cross-linguistic limitations.

### 4.2 Performance of models in the ophthalmic clinical assistance scenario

All evaluations demonstrated high concordance except for moderate agreement (κ = 0.310) in accuracy grading for the ocular surface diseases subgroup. Further analysis revealed a systematic rating bias in this subgroup, where 87 out of 90 accuracy gradings were classified as *“Good”* and 3 as “*Borderline,”* suggesting the extreme distribution pattern of the categorical data likely influenced the observed concordance metrics.

In accuracy evaluations across ophthalmic clinical assistance scenarios, no model received a *“Poor”* grade, indicating none of the generated texts were deemed potentially harmful to patients in this assessment framework. Gemini 1.5 Flash consistently outperformed other models in both overall accuracy gradings and completeness scores. Although the effect size of accuracy grading (Cramér’s V = 0.183) was small to moderate. It remains clinically meaningful given that all evaluated models represent LLMs with already high baseline performance. In particular, the 20.7% difference in positive feedback between Gemini 1.5 Flash and iFLY Healthcare (φ = 0.226) could substantially influence the quality of clinical assistance. Compared with the accuracy grading, completeness scores exhibited a larger effect size (η^2^ = 0.154). The observed contrast suggests that differences among LLMs are more evident in how comprehensively they construct responses than in how precisely they convey factual content. This may reflect that variations in information integration and expression capabilities across models exceed those in their underlying medical knowledge bases.

This study further conducted readability assessments to compare linguistic characteristics of generated texts across models. Effect size analysis revealed R2 as the most discriminative metric. Claude 3 Opus produced texts with a shorter average sentence length and the lowest proportion of adverbs and conjunctions, demonstrating significantly superior overall readability among all models. These findings suggest that its output featured more concise sentence structures, thereby enhancing the readability of clinical support texts.

### 4.3 Performance of models in the ophthalmic disease diagnosis and treatment scenario

#### 4.3.1 Overall and subspecialty performance of LLMs

This section evaluated LLMs’ ophthalmic knowledge mastery and clinical reasoning through simulated intermediate technical qualification examinations. Claude 3 Opus achieved superior overall performance with 81.07% accuracy, outperforming other models. Although overall effect sizes were small (Cramér’s V = 0.115), subgroup analysis revealed substantial variations across knowledge domains. Notably, Claude 3 Opus excelled in the Basic Knowledge subject (Subject 1) and the Professional Practice Ability subject (Subject 4). Simultaneously, Gemini 1.5 Flash demonstrated particular strength in the Related Professional Knowledge subject (Subject 2). iFLY Healthcare showed a relative advantage in the Professional Knowledge subject (Subject 3), though it did not show statistical significance among models. The absence of significant differences in Subject 3 suggests that current LLMs have reached comparable proficiency levels in core professional ophthalmic knowledge.

Across four examination modules, LLMs attained the highest mean accuracy in the Related Professional Knowledge subject (Subject 2, 77.40%) and the lowest in the Professional Practice Ability subject (Subject 4, 61.33%). Subject 2 primarily assessed interdisciplinary knowledge integration (internal medicine, neurology, imaging, genetics), highlighting LLMs’ superior capabilities in big data processing and cross-domain knowledge synthesis. Gemini 1.5 Flash (56%) and iFLY Healthcare (49.33%) failed to meet passing thresholds in Subject 4, which employed case-based multiple-select questions requiring temporal/spatial reasoning, clinical progression analysis, and evidence-based decision-making. The performance deficit in this module may stem from the inherent complexity of clinical scenarios and the multiple-response format.

#### 4.3.2 ChatGPT 4’s performance on standardized qualification examinations across languages

Cross-linguistic comparisons demonstrated ChatGPT 4’s accuracy (65.07%) in this Chinese ophthalmic assessment trailed its performance in US-based ophthalmic question banks (70%–80%) (Cai et al., 2023; Teebagy et al., 2017; Taloni et al., 2023; Mihalache et al., 2023; Antaki et al., 2024), yet surpassed Japanese ophthalmology board examination benchmarks (46.2%) (Sakai et al., 2023), indicating significant language-dependent performance variations.

#### 4.3.3 Hallucinated content and factual inaccuracies in models’ responses

Although the models demonstrated strong performance in simulated intermediate-level ophthalmic qualification examinations and other professional certification tests, certain generated content during testing may contain erroneous medical information that could potentially mislead patients and result in severe consequences. This phenomenon of generating incorrect or misleading content in medical responses is termed “hallucination” (Lee et al., 2023; Nori et al., 2023). For instance, five models committed identical errors while providing seemingly plausible explanations when responding to a clinical question in the Professional Knowledge subject (Subject 3).

The case description was as follows: The patient is a 25-year-old female. Since birth, the patient’s right eye has opened smaller than the left eye, and there is no morning or evening lightness, which is not related to chewing. Examination results: Vou 1.2, right eyelid fissure height 8 mm, upper face edge covers the upper corneal edge 2 mm, levator palpebrae superioris muscle strength 9 mm. Left eyelid fissure height 10.5 mm, upper eyelid edge is 0.5 mm above the upper corneal edge, levator palpebrae superioris muscle strength 10 mm, no incomplete eyelid closure, no exophthalmos. No diplopia, all systemic examinations are regular.

However, the LLMs were confused by the description of “right eye opening smaller than left eye since birth” in the question and mistakenly regarded the right eye as the affected eye, overlooking critical information provided in the question, such as the height of the palpebral fissure and the distance between the upper eyelid edge and the corneal edge. All models selected option A, congenital ptosis of the right eye. However, the affected eye should be the left eye, and the correct answer should be option D, congenital eyelid retraction of the left eye. The model failed to thoroughly compare and evaluate the binocular data (levator muscle strength and the measurement of the upper eyelid margin covering the corneal limbus), resulting in a misjudgment and further misinterpretation.

The research findings demonstrate that ophthalmic diagnosis and management require not only domain-specific knowledge reserves but, more critically, rigorous logical reasoning capabilities and multi-source data integration competencies. Although all LLMs evaluated in this study passed the simulated intermediate-level ophthalmic health technology qualification examination, preliminarily validating their clinical applicability, persistent issues of “hallucination” and factual inaccuracies remain prevalent in generated content. Such deficiencies risk misleading clinical decision-making processes and potentially compromising patient safety. Future advancements should focus on targeted model training incorporating ophthalmic clinical expertise and diagnostic reasoning frameworks to facilitate the substantive transition of LLMs from “medical information generators” to “trustworthy decision-support systems.”

### 4.4 Performance of models in the ophthalmologist-patient communication assistance scenario

**FIGURE 4:**
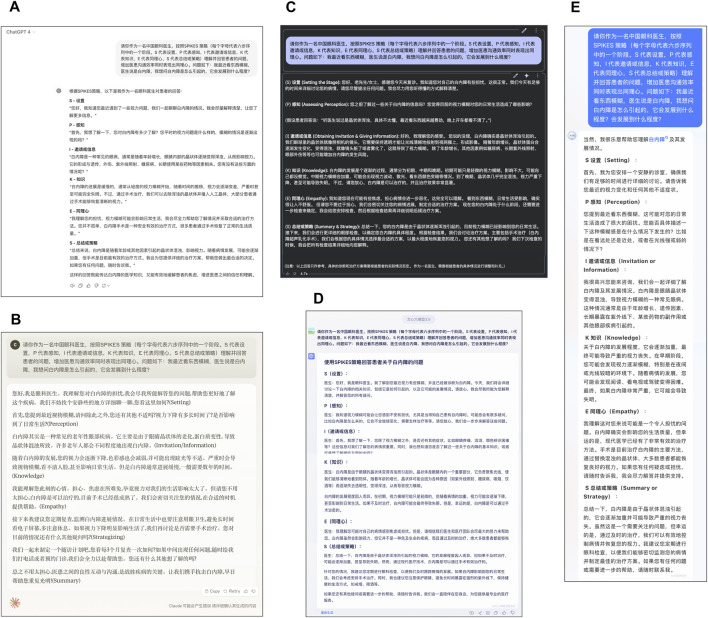
Presentation of large language models generates responses for physician-patient interaction. **(A)** ChatGPT 4, **(B)** Claude 3 Opus, **(C)** Gemini 1.5 Flash, **(D)** ERNIE 3.5, **(E)** iFLY Healthcare.

All LLMs generated physician-patient communication texts with elevated empathy levels when provided with specific input prompts. Responses across models frequently contained phrases such as “I understand your concerns,” “I will make every effort to assist you,” and “Let’s work together,” (Figure 4). Conventional evaluation metrics have primarily focused on accuracy in responding to ethical/moral dilemma test questions (Brin et al., 2023) and simplistic ratings of response empathy levels (Ayers et al., 2023; Yeo et al., 2023). This investigation employed natural language processing techniques with two Chinese textual analysis databases (Cnsenti and Cntext-2.1.3) for sentiment analysis and emotion/socialness attribute assessment of model-generated responses. The methodology enabled quantitative evaluation of LLMs’ emotional regulation capabilities and relation-building competence in clinical communication scenarios.

In emotional generation capacity, no significant inter-model differences emerged in positive-to-negative sentiment ratios (Pos/Neg) or emotional dimension values (Emotion), with negligible effect sizes. This suggests that all evaluated models have similar basic abilities in emotional expression. In contrast, socialness attributes revealed substantial differences (η^2^ = 0.396, large effect). Gemini 1.5 Flash model achieved the highest positive-to-negative sentiment ratio (Pos/Neg) and Emotion dimension values, though inter-model differences were statistically modest. Regarding Socialness attributes, Gemini 1.5 Flash and ERNIE 3.5 demonstrated superior performance compared to ChatGPT 4, Claude 3 Opus, and iFLY Healthcare, with large effect sizes. These effect magnitudes indicate practical significance beyond statistical significance, potentially translating to meaningful improvements in patient engagement and therapeutic alliance formation in clinical settings. Collectively, the Gemini 1.5 Flash-generated communication assistant texts demonstrated optimal performance across emotional tendency, socialness, and emotion metrics, empirically validating its advanced abilities in emotional modulation and the construction of therapeutic alliances through textual outputs.

### 4.5 Mechanistic analysis of model performance variations

The performance variations observed among LLMs may reflect fundamental differences in model architectures, training methodologies, and data characteristics. The model-specific strengths identified in our study can be potentially explained through the following mechanisms.

Gemini 1.5 Flash’s superior performance in doctor-patient communication scenarios may be associated with its advanced multimodal architecture and training strategies. The model employs a Transformer-decoder architecture pretrained on multimodal and multilingual datasets encompassing web documents, code, images, audio, and video content, with an extended context processing capability of up to 10 million tokens (Team et al., 2024). Such multimodal learning may enable the model to recognize emotional semantics and social cues across modalities, facilitating superior sensitivity to affective and contextual nuances in human communication. Consequently, Gemini 1.5 Flash achieved the highest scores in both Emotion and Socialness dimensions, suggesting enhanced empathetic expression and relationship-building capabilities.

Claude 3 Opus’s proficiency in clinical reasoning tasks may stem from its distinctive “Constitutional AI” training framework (Anthropic, 2025). This paradigm integrates self-supervised learning, value learning, and reinforcement learning from human feedback (RLHF) to enforce logical consistency and ethical alignment, which may help ensure outputs are safe, helpful, and honest. The Professional Practice Competency (Subject 4) of the ophthalmology qualification examination inherently requires rigorous evidence-based medical judgment and ethical decision-making abilities. Claude 3 Opus’s training mechanism emphasizes step-by-step logical analysis and adherence to ethical principles, which may enable it to integrate knowledge more effectively and construct logically rigorous clinical reasoning chains when processing complex ophthalmic cases. Furthermore, the model’s demonstrated excellence in reasoning, mathematical, and coding benchmarks, coupled with its larger training corpus, may provide foundational support for clinical knowledge integration.

ChatGPT 4 presented a balanced performance across all evaluated dimensions, likely reflecting the combined effects of its iterative pretraining on large-scale corpora and optimized fine-tuning algorithms (OpenAI, 2025; Bubeck et al., 2023), with potential strengths in multimodal processing relevant to ophthalmology imaging analysis. Compared to GPT-3.5, GPT-4 demonstrated a 40% improvement in generating valid responses and an 82% reduction in inappropriate responses, which may demonstrate the effectiveness of architectural refinements and alignment procedures.

For Chinese language processing, distinct strengths emerged. ERNIE’s continuous pretraining framework and extensive Chinese corpus training may contribute to its performance in handling Chinese medical terminology and communication patterns (Research, 2025). iFLY Healthcare’s exceptional performance in medical-specific tasks may underscore the importance of domain-specific training data, with its trillion-token medical literature database potentially providing substantial ophthalmology-relevant knowledge (Healthcare, 2023). Furthermore, its consumer-facing application further integrates this domain knowledge by automatically generating individual electronic health records from unstructured patient dialogue. This highlights the potential of LLM-driven tools to streamline medical documentation and bridge the gap between conversational AI and real-world clinical workflows.

These findings collectively highlight the value of domain-specific pretraining, linguistic localization, and application-oriented integration in advancing medical LLMs toward practical deployment in healthcare.

### 4.6 Limitations and future directions

This study represents a pioneering effort to simulate and evaluate the performance of LLMs across three common clinical scenarios in ophthalmology. However, several limitations merit consideration and should inform future research.

First, although LLMs demonstrated competent performance in clinical assistance and diagnostic simulation tasks, these findings cannot be directly extrapolated to suggest parity with human ophthalmology attending physicians in real-world practice. Notably, LLMs exhibited divergent performance when addressing insurance-related inquiries in the ophthalmologist-patient communication assistance scenario. Several key factors unaddressed in current simulations include regional variations in healthcare policies, geographical disparities in medication availability, and economic differences among treatment options.

Second, the natural language assessment tools have some limitations. Readability analyses based solely on surface-level textual features (e.g., sentence length, syntactic complexity) are insufficient to capture deeper aspects of text quality, such as logical coherence, information architecture, and alignment with the reader’s knowledge base. Nevertheless, our study employed the readability module of Cntext-2.1.3, which offers concise, efficient, and empirically validated indicators that can intuitively reflect the linguistic complexity of Chinese clinical texts. Its interpretable metrics (average sentence length, frequency of adverbs and conjunctions, and the Fog Index) are particularly suitable for cross-model comparison of large language model outputs. Future work may further enhance this approach by incorporating more advanced frameworks, such as AlphaReadabilityChinese (Lei et al., 2024), which integrate lexical, syntactic, and semantic entropy features for a multidimensional evaluation of linguistic and cognitive difficulty. Similarly, sentiment analysis tools may lack the resolution needed to detect nuanced emotional expressions, such as irony, ambivalence, or context-specific affective cues.

Despite these limitations, this study provides valuable insights into the capabilities and constraints of LLMs in typical ophthalmic practice scenarios. Future work should incorporate authentic clinical case data and develop more comprehensive, context-aware evaluation frameworks to address current methodological constraints. These refinements will be essential for advancing the reliable and responsible integration of LLMs into ophthalmology and broader clinical practice.

## 5 Conclusion

This study innovatively simulated three clinical application scenarios for LLMs in ophthalmic practice: ophthalmic clinical assistance, ophthalmic disease diagnosis and treatment, and ophthalmologist-patient communication. We systematically assessed the text generation abilities of major LLMs in specialized ophthalmic clinical areas. The results demonstrate that Gemini 1.5 Flash exhibits significant advantages in delivering accurate and comprehensive ophthalmic clinical support information, particularly demonstrating exceptional emotional regulation capabilities and relationship-building competence in doctor-patient communication scenarios. Claude 3 Opus generates clinical support materials with superior readability and demonstrates stronger ophthalmic knowledge integration capacity and clinical reasoning ability in simulated examinations.

These findings reveal the tremendous potential of LLMs in ophthalmic applications while providing scientific evidence for ophthalmologists to select optimal artificial intelligence models in clinical practice. Future directions should concentrate on developing more sophisticated evaluation frameworks to guide continuous optimization of medical language models, ultimately promoting intelligent development in ophthalmic medicine.

## Data Availability

The original contributions presented in the study are included in the article/Supplementary Material, further inquiries can be directed to the corresponding author.
