# Supplementary material for: Evaluating the competence of large language models in ophthalmology clinical practice: a multi-scenario quantitative study
Source: Front Cell Dev Biol. 2025 Dec 2;13:1704762. doi: 10.3389/fcell.2025.1704762 (PMC12705536; doi:10.3389/fcell.2025.1704762)
Supplement: Supplementary file 1 [file Table1.docx]

Table S1. Chinese Ophthalmic Guidelines and Expert Consensuses Used for Question Design

| No. | Title of Guideline or Consensus | Year |
| --- | --- | --- |
| 1 | Expert Consensus on the Classification and Treatment of Retinopathy of Prematurity in China | 2023 |
| 2 | Chinese Expert Consensus on the Diagnosis and Treatment of Intraocular Foreign Bodies | 2021 |
| 3 | Chinese Expert Consensus on the Diagnosis and Treatment of Orbital Foreign Bodies | 2021 |
| 4 | Chinese Expert Consensus on the Diagnosis and Management of Infectious Endophthalmitis after Ophthalmic Surgery | 2022 |
| 5 | Chinese Expert Consensus on the Prophylaxis and Management of Traumatic Infectious Endophthalmitis | 2023 |
| 6 | Chinese Expert Consensus on Diagnosis and Treatment of Diabetic Optic Neuropathy | 2022 |
| 7 | Expert Consensus on Clinical Diagnosis and Treatment Path of Retinal Vein Occlusion in China | 2024 |
| 8 | Chinese Glaucoma Guidelines | 2020 |
| 9 | Evidence-based Guidelines for Diagnosis and Treatment of Age-related Macular Degeneration in China | 2023 |
| 10 | Chinese Expert Consensus on Endoscopic Dacryocystorhinostomy for Chronic Dacryocystitis | 2020 |
| 11 | Chinese Guideline on the Diagnosis and Treatment of Thyroid-associated Ophthalmopathy | 2022 |
| 12 | Chinese Expert Consensus on Dry Eye: Treatment | 2020 |
| 13 | Expert Consensus on Prevention and Treatment of Amblyopia in Children | 2021 |
| 14 | Chinese Expert Consensus on the Perioperative Management of Cataracts in Children | 2022 |
| 15 | Chinese Expert Consensus on the Diagnosis and Treatment of Herpes Simplex Keratitis | 2023 |
| 16 | Chinese Guideline for Cataract Surgery in Adults | 2023 |
| 17 | Evidence-based Guidelines for Diagnosis and Treatment of Diabetic Retinopathy in China | 2022 |
| 18 | Chinese Expert Consensus on Corneal Transplantation | 2015 |
| 19 | Guidelines for Diagnosis and Treatment of Syphilis, Gonorrhea and Genital Chlamydia trachomatis Infection | 2020 |
| 20 | Expert Consensus on Myopia Management White Paper | 2022 |
| 21 | Expert Consensus on the Application of Spectacles Related to Myopia Prevention and Control in Myopia Management | 2023 |

Table S2. Patient-physician communication question distribution

| Options | Respondents | Proportion |  | The quantity of test questions in scenario |
| --- | --- | --- | --- | --- |
| Disease mechanism and diagnostic processes | 11 | 18.33% |  | 4 |
| Disease treatment limitation, adverse risk and prognosis | 22 | 36.67% |  | 7 |
| Impact of disease and alleviating patients' negative emotions | 5 | 8.33% |  | 2 |
| Cost and insurance policy of disease treatment | 22 | 36.67% |  | 7 |
